# Deformation of the moving magnetic skyrmion lattice in MnSi under electric current flow

**DOI:** 10.1038/s42005-019-0175-z

**Published:** 2019

**Authors:** D. Okuyama, M. Bleuel, J.S. White, Q. Ye, J. Krzywon, G. Nagy, Z.Q. Im, I. Živković, M. Bartkowiak, H.M. Rønnow, S. Hoshino, J. Iwasaki, N. Nagaosa, A. Kikkawa, Y. Taguchi, Y. Tokura, D. Higashi, J.D. Reim, Y. Nambu, T.J. Sato

**Affiliations:** 1 Institute of Multidisciplinary Research for Advanced Materials (IMRAM), Tohoku University, Katahira 2-1-1, Sendai 980-8577, Japan.; 2 NIST Center for Neutron Research, National Institute of Standards and Technology, 100 Bureau Drive, Gaithersburg, MD 20899-8562, USA.; 3 Department of Materials Science and Engineering, University of Maryland, College Park, MD 20742-2115, USA.; 4 Laboratory for Quantum Magnetism, Institute of Physics, École Polytechnique Fédérale de Lausanne (EPFL), CH-1015 Lausanne, Switzerland.; 5 Laboratory for Neutron Scattering and Imaging (LNS), Paul Scherrer Institut (PSI), CH-5232 Villigen, Switzerland.; 6 Laboratory for Scientific Developments and Novel Materials (LDM), Paul Scherrer Institut (PSI), CH-5232 Villigen, Switzerland.; 7 RIKEN Center for Emergent Matter Science (CEMS), Wako 351-0198, Japan.; 8 Department of Applied Physics, University of Tokyo, Tokyo 113-8656, Japan.; 9 Institute for Materials Research (IMR), Tohoku University, Katahira 2-1-1, Sendai 980-8577, Japan.

## Abstract

Topological defects are found ubiquitously in various kinds of matter, such as vortices in type-II superconductors, and magnetic skyrmions in chiral ferromagnets. While knowledge on the static behavior of magnetic skyrmions is accumulating steadily, their dynamics under forced flow is still a widely open issue. Here, we report the deformation of the moving magnetic skyrmion lattice in MnSi under electric current flow observed using small-angle neutron scattering. A spatially inhomogeneous rotation of the skyrmion lattice, with an inverse rotation sense for opposite sample edges, is observed for current densities greater than a threshold value *j*_t_ ~ 1 MA m^−2^ (10^6^ A m^−2^). Our result show that skyrmion lattices under current flow experience significant friction near the sample edges due to pinning, this being a critical effect that must be considered for anticipated skyrmion-based applications at the nanoscale.

Topological defects, i.e., defects that cannot be annihilated by continuous deformation, are found ubiquitously in various kinds of matter, such as screw dislocation in crystals^1–3^, defect in nematic liquid crystal^4–6^, and quantum vortices in superfluid and type-II superconductor^7–13^. A magnetic skyrmion, one of such topological defects, is a swirling spin texture characterized by a discrete topological number, called the skyrmion number. Such topologically nontrivial states of matter were first theoretically predicted in nonlinear field theory^14,15^, and subsequently in chiral magnets^16^. Experimentally, magnetic skyrmions often condense into triangular-lattice, observed as sixfold magnetic Bragg reflections in small-angle neutron scattering (SANS), first discovered by Mühlbauer et al. in the prototypical chiral magnet MnSi^17^. To date, triangular skyrmion-lattice structures are widely confirmed in various magnets ranging from metallic to insulating compounds^18–23^, and also by various techniques such as Lorentz transmission electron microscopy (TEM) and magnetic force microscopy^24,25^.

There are several prominent characteristics of magnetic skyrmions that make them quite intriguing for fundamental, as well as technological application viewpoints. One is its topological protection; once created, the skyrmion can hardly be annihilated^18^. Hence, there is a possibility for the magnetic skyrmion to be used as a robust information storage/carriage unit in future spintronics devices. In metallic skyrmion compounds, there is another important characteristic, namely its strong coupling to electric current flow. The electric current density required to realize the skyrmion-lattice motion is remarkably small at *j* ~ 1 MA m^−2^ (10^6^ A m^−2^), compared to that required to move magnetic domain boundaries in conventional magnetic materials *j* ≥ 1 GA m^−2^ (10^9^ A m^−2^)^26–29^. The potential for controlling magnetic skyrmions with such a low current density, together with their topologically protected nature, makes spintronics application promising^30^. Hence, magnetic skyrmions continue to attract strong attention, and remain under intense scrutiny for elucidating their dynamical behavior under electric current.

A number of reports have appeared to date on the motion of magnetic skyrmions under electric current flow. In the pioneering study of Jonietz et al. using SANS, a rotation of the skyrmion lattice in MnSi was detected for an electric current density greater than the threshold value *j*_t_ ~ 1 MA m^−2 31^. There, it was argued that the electric current flow alone introduces translational motion only via spin transfer torques, which cannot be detected by SANS. Hence, a thermal gradient was intentionally applied in their experiment, which provides spatial inhomogeneity of the spin transfer torque. Indeed, the skyrmion-lattice rotation was observed only when both the current flow and thermal gradient were present. Nonetheless, by careful examination of the presented data, it can be seen that the skyrmion-lattice peaks are significantly broadened under the electric current flow, the origin of which was not discussed.

Skyrmion-lattice motion under thermally homogeneous conditions was studied in the other works. From Hall-resistivity measurements on MnSi, the skyrmions move in the direction of the electric current above a critical current density *j*_t_ ~ 0.5 MA m^−2^, while the emergent electric field is transverse to the current^32,33^. From a Lorentz TEM study of FeGe, the skyrmion lattice was observed to ‘disappear’ as the electric current exceeded a threshold value, indicating the skyrmion lattice to move much faster than the Lorentz TEM time frame^34^. These studies show that the electric current flow induces the skyrmion-lattice motion. However, important microscopic information, such as the skyrmion-lattice deformation, under thermally homogeneous conditions remains largely unexplored.

Here, we investigate the deformation of the moving magnetic skyrmion lattice under an electric current flow using the SANS technique, paying careful attention towards keeping any thermal gradient as small as experimentally achievable. We observe clearly a broadening of the skyrmion-lattice-reflection peaks for *j* > *j*_t_, indicating the skyrmion lattice to deform considerably when the lattice starts to flow translationally. We further find that the peak broadening is due to a spatially inhomogeneous rotation of the skyrmion lattice; by measuring opposing-sample edges, we observed a counter-rotating behavior of the skyrmion lattice. The observed rotation appears to be not continuous but fixed angle in the SANS measurement time frame (20 min). While the rotation direction does not change under inversion of the external magnetic field, it does revert when the electric current direction is reversed. Finally, the peak broadening and corresponding lattice rotation remain finite even after the current is turned off, indicating a plastic deformation of the skyrmion lattice under the force exerted by the electric current.

## Results

### Peak broadening of skyrmion reflection above *j*_t_.

**Fig. 1:**
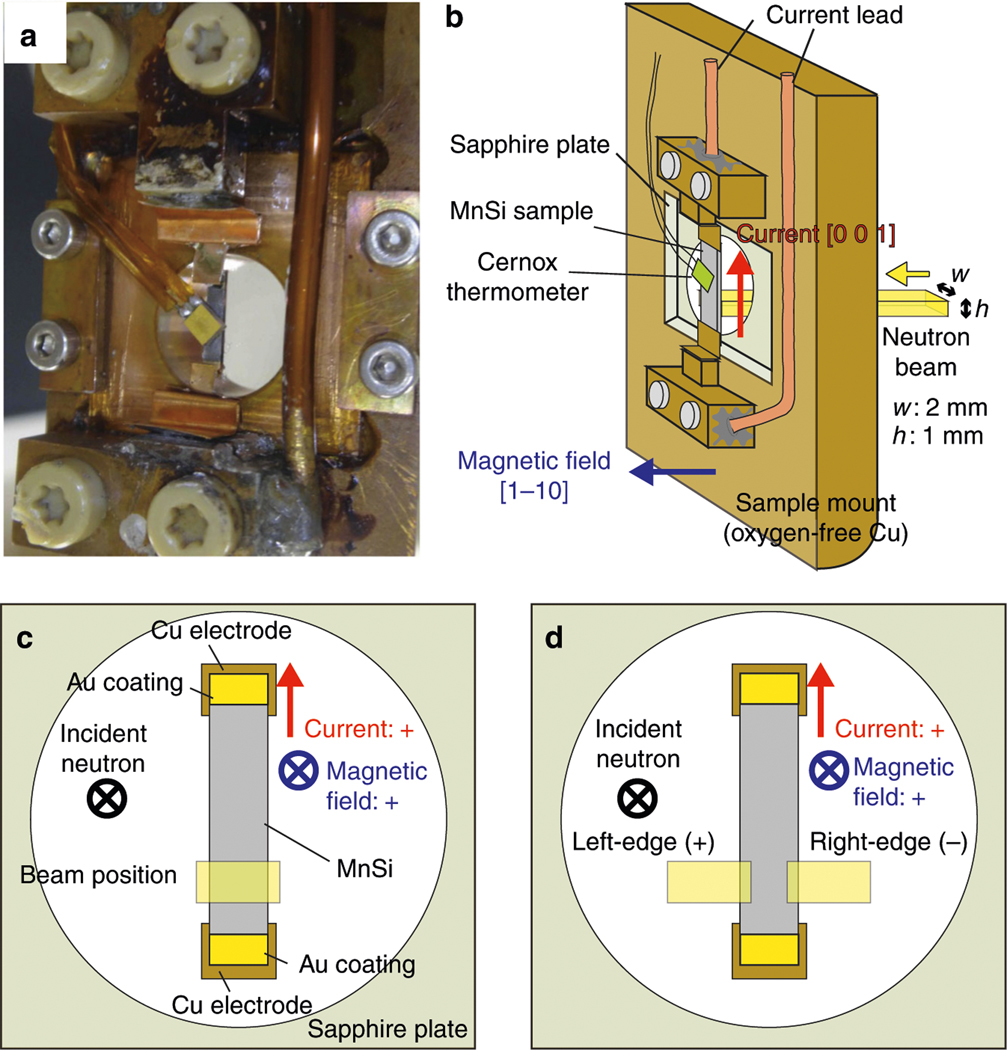
Experimental setting of small angle neutron scattering (SANS) with keeping any thermal gradient as small as experimentally achievable. **a**, **b** Photograph (**a**) and schematic drawing (**b**) of the MnSi sample and sample mount for the NG7 experiment. The incident neutron beam was shaped to be 2.0 mm (width) × 1.0 mm (height) by an aperture. c, d Schematic illustration of the beam illumination area for investigating the peak broadening (**c**) and counter-rotating behavior at the sample edges (**d**) of the skyrmion-lattice peaks

**Fig. 2:**
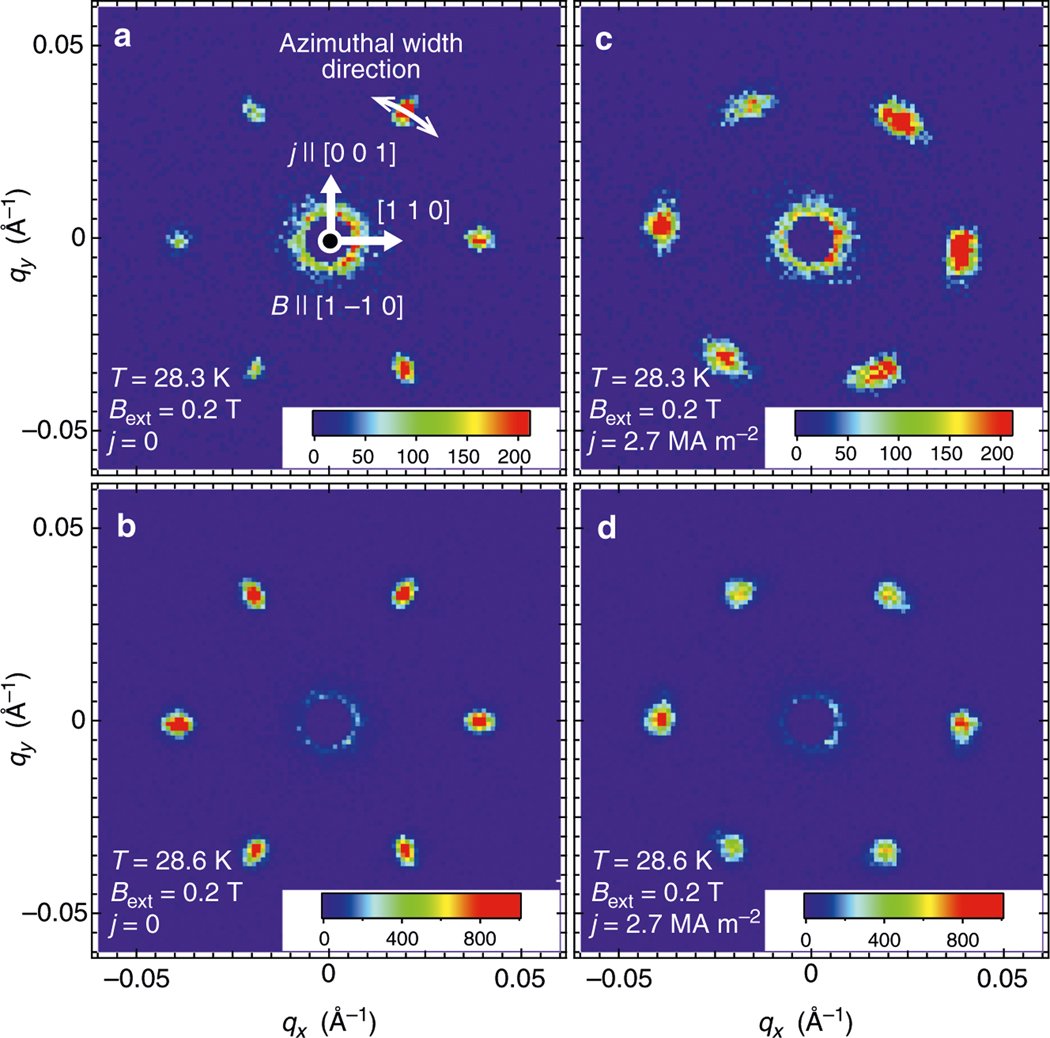
Small angle neutron scattering (SANS) data for investigating the peak broadening of the skyrmion reflection. **a–d** SANS measured at *B*_ext_ = 0.2 T. The skyrmion reflections were measured at *T* = 28.3 K (28.6 K) under *j* = 0 (2.7 MA m^−2^), respectively. All data were measured for 10 min. When the intensities of the six skyrmion reflections are not equivalent, the sample is slightly misoriented away from the Bragg position. The azimuthal width direction is indicated by the arrow in panel **a**. *q*_*x*_ and *q*_*y*_ stand for the reciprocal lattice space directions

Representative SANS patterns measured at NG7 SANS instrument using the experimental setting in Fig. 1 are shown in Fig. 2a–d. They were measured by imaging an entire horizontal cross-section of the sample, as shown in Fig. 1c. A photograph of the sample mount and its schematic illustration are shown in Fig. 1a, b, respectively. Figure 2a, b show the skyrmion reflections observed at the two temperatures, *T* = 28.3 K and 28.6 K, respectively, under *B*_ext_ = 0.2 T and *j* = 0. The six-fold magnetic reflections characteristic of the skyrmion lattice were observed in a temperature range of 28 K < *T* < 29.2 K at *B*_ext_ = 0.2 T. These observations are identical to those in the earlier works^17,31^.

Figure 2c, d show the SANS patterns obtained at *j* = 2.7 MA m^−2^ and *B*_ext_ = 0.2 T. By comparing with the zero-current-flow counterparts in Fig. 2a, b, a considerable azimuthal broadening of the skyrmion-lattice peaks was observed. The peak broadening is temperature dependent at *j* = 2.7 MA m^−2^; the width is considerably larger at *T* = 28.3 K compared to that at *T* = 28.6 K. This result clearly indicates the significant deformation of the skyrmion lattice under large electric current. In stark contrast, no intrinsic peak broadening in radial direction was observed under the electric current.

**Fig. 3:**
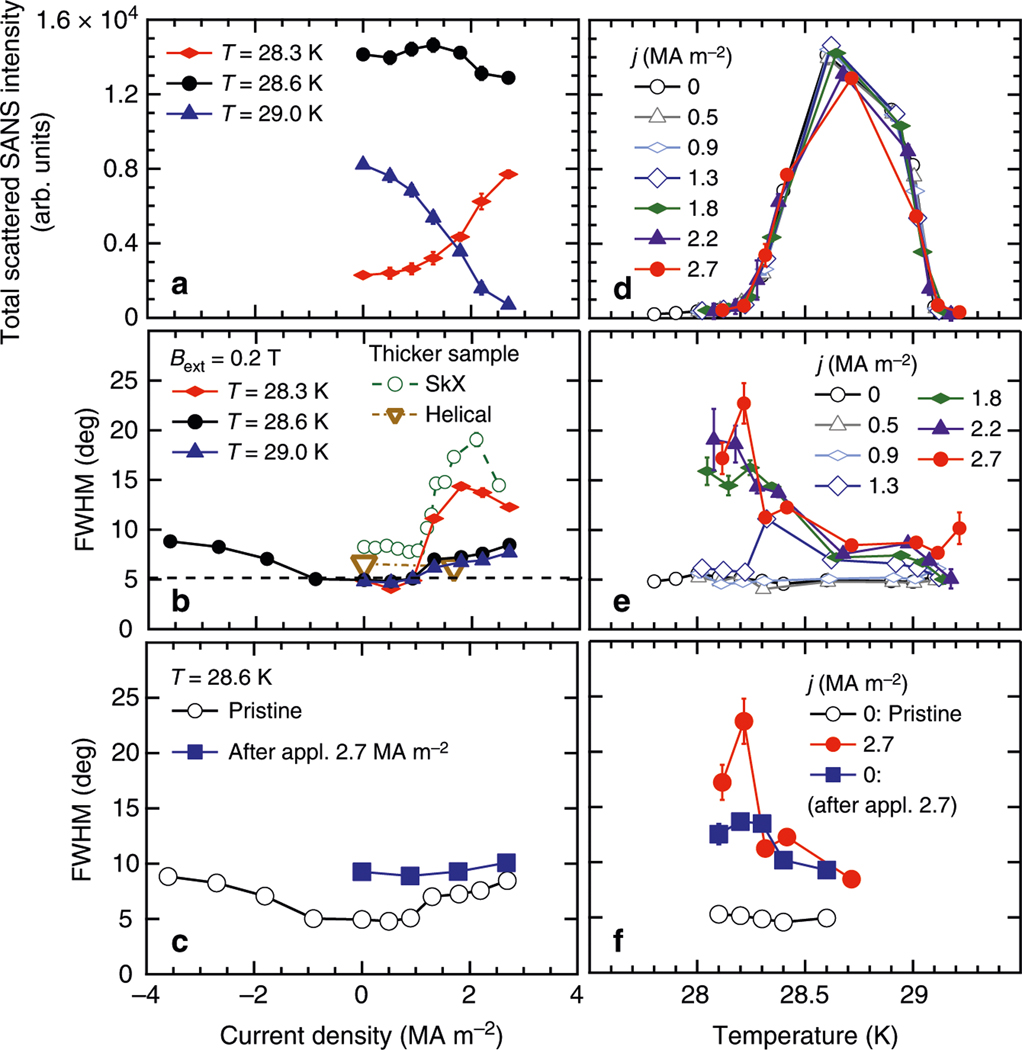
Current-density and temperature dependences of the skyrmion reflections for investigating the peak broadening of the skyrmion reflection. **a–c**, Current-density dependences of the total scattered small angle neutron scattering (SANS) intensity (**a**) and the azimuthal spot widths (**b** and **c**) of the skyrmion reflections measured at three temperatures. Here, we use 1σ standard deviation as error bar. The temperature used in **a–c** is the sensor temperature, and therefore not the true temperature estimated from the ordering temperature of the helical phase. Thus, the increase (decrease) of the intensity at T = 28.3 K (29.0 K and 28.6 K) under the high electric current is due to sample heating. For the measurement of the negative current density, we reset the peak broadening of the skyrmion reflection at zero current density once by heating the sample above the phase transition temperature. In **a** and **c–f**, data are measured at *B*_ext_ = 0.2 T. In **b** the azimuthal width of the skyrmion (*B*_ext_ = 0.2 T) and helical (*B*_ext_ = 0) reflections at *T* = 28.6 K measured by the thicker sample are also shown. **d–f** Temperature dependences of the total scattered SANS intensity (**d**) and azimuthal widths (**e**, **f**) of the skyrmion reflections at each current density. In **c** and **f** additionally, the dependence of the width after applying *j* = 2.7 MA m^−2^ is shown. The broadening of the skyrmion reflection persisted for more than one hour after the applied current density was removed

To examine quantitatively the current dependence of the skyrmion-lattice peaks, in Fig. 3a, b, we show, respectively, the total scattered SANS intensity and azimuthal spot widths as functions of current density at three selected temperatures and *B*_ext_ = 0.2 T. The spot width remains almost constant in the low current density region (*j* < *j*_t_ ~ 1 MA m^−2^) for all measured temperatures. At *T* = 28.3 K, the width shows a steep increase for *j* > *j*_t_. This clearly confirms the existence of a threshold current density for skyrmion-lattice deformation. It may be noted that the value of *j*_t_ is in good agreement with the current density above which skyrmion-lattice motion was detected in the earlier studies^31,32^. Hence, the peak broadening detected above *j*_t_ in the present study is a direct consequence of skyrmion-lattice motion driven by the electric current. In stark contrast to the skyrmion-lattice peaks, we observed no peak broadening for the reflections in the helical phase at *B*_ext_ = 0 up to 1.67 MA m^−2^, as shown by the open triangular symbol in Fig. 3b.

### Plastic deformation of skyrmion lattice.

Once induced by the electric current, the broadening of the skyrmion-lattice peaks persists, even after the electric current is removed. In Fig. 3c, starting from the pristine state, the width increases monotonically up to *j* = 2.7 MA m^−2^ above *j*_t_, whereas when lowering the current, the width stays almost the same down to *j* = 0. This clearly indicates that the skyrmion lattice remembers the deformation generated in the driven state. In other words, the skyrmion lattice locally deforms like plastic matter when driven under electric current. This plastic deformation may be due to impurity pinning.

Figure 3d, e show the temperature dependences of, respectively, the total scattered SANS intensity and azimuthal spot widths at fixed current density. At *j* = 2.7 MA m^−2^, the width shows a significant temperature dependence below 28.5 K; it monotonically increases as temperature is decreased. Below *j*_t_, the width shows negligible temperature dependence. Figure 3f shows the temperature dependence of the width at *j* = 0, measured with decreasing temperature, after having applied the current density 2.7 MA m^−2^ at *T* = 28.6 K. Independent of the temperature, the spot widths remain broadened compared with the pristine state at *j* = 0 after the current ramping, confirming the robust memory effect for the skyrmion-lattice deformation.

### Spatially inhomogeneous rotation of skyrmion lattice.

**Fig. 4:**
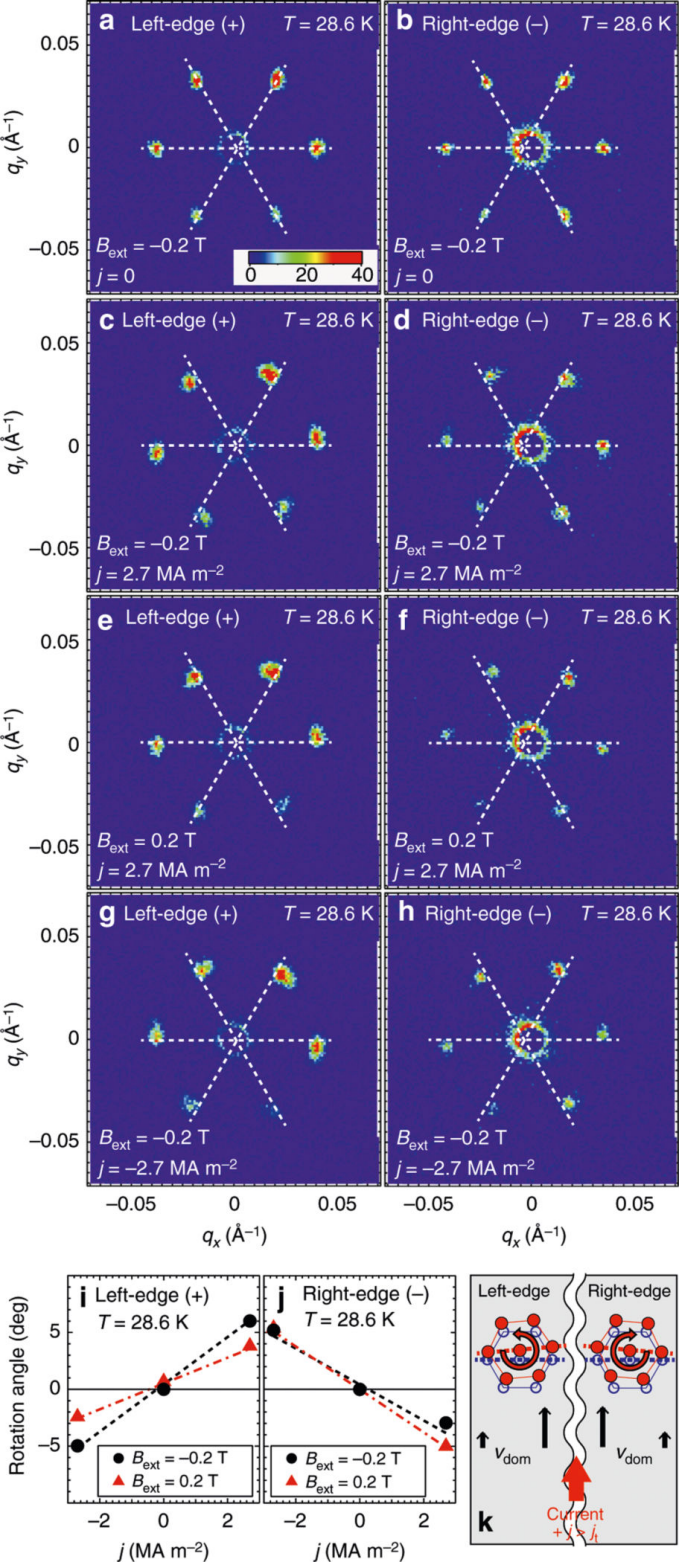
Small angle neutron scattering (SANS) data for investigating counter-rotating behavior at the sample edges of the skyrmion-lattice peaks. SANS measured from the left-edge (+) or right-edge (−) of the sample. **a–h**, The skyrmion reflections at *T* = 28.6 K, respectively, measured at *B*_ext_ =−0.2 (0.2 T), *j* = 0 (2.7 MA m^−2^), and at the left-edge (right-edge). All data were measured for 20 minutes. Dashed lines are guides to eye for the peak positions of the skyrmion reflections in pristine condition. **i**, **j** The current-density and magnetic-field dependences of the averaged rotation angles of the skyrmion reflections at *T* = 28.6 K at left-edges and right-edges. The circle and triangle denote the data at *B*_ext_ =−0.2 and 0.2 T, respectively. The dashed lines are guides to eye. **k** Schematic illustration of the skyrmion-lattice rotation above j_t_. Blue and red circles stand for the pristine and moved skyrmions, respectively. Black arrows and *v*_dom_ explain the velocity of the skyrmion-lattice domain near the sample edges

To investigate the origin of the broadening of the skyrmion reflections, we performed SANS measurements on the left-edge (+) or right-edge (−) of the sample, as shown schematically in Fig. 1d. The size of the neutron illumination area is approximately 0.2 mm (width) × 1.0 mm (height). The SANS patterns obtained at *T* = 28.6 K and *B*_ext_ =−0.2 T without electric current are shown in Fig. 4a, b for the left-edge and right-edge, respectively. The observed patterns are identical for the two edges, and in good agreement with the data shown in Fig. 2b. In marked contrast to the zero-current condition, under *j* = 2.7 MA m^−2^ the reflection patterns taken from the left-edge and right-edge parts exhibit counterclockwise and clockwise rotations, respectively, as shown in Fig. 4c, d.

Next, the effect of inverting the current and magnetic-field directions was investigated. Figure 4e, f show data taken under an inverted magnetic field *B*_ext_ = 0.2 T. Apparently, the rotation direction does not change by inverting *B*_ext_. On the other hand, the inversion of the current direction (from *j* =+2.7 to −2.7 MA m^−2^) results in a sign change of the rotation angle, as clearly seen by comparing Fig. 4g, h (*j* =−2.7 MA m^−2^) with Fig. 4c, d (*j* =+2.7 MA m^−2^). This indicates that the skyrmion-lattice rotation observed in the thermally homogeneous condition depends not on the magnetization direction, but only on the current direction. It should be noted that the transverse translational motion is due to the scalar spin chirality, which depends on the third order of the magnetization density $\vec{M}(\vec{R})$, whereas the longitudinal translational mode along the electric current is due to the dissipative tensor which is in the second order in $\vec{M}(\vec{R})$^35,36^. The independence of the observed rotation angle to the magnetization direction thus indicates that the rotation effect should be attributed to the longitudinal translational motion of the skyrmions under the electric current. In the earlier study of Jonietz et al., the rotation of the magnetic skyrmion lattice was found under the electric current flow in the thermal gradient^31^. Zhang et al. recently found the continuous rotation of the magnetic skyrmion lattice by applying only the magnetic field gradient^37^. In these two studies, the gradient of either temperature or magnetic field is the key to induce skyrmion lattice rotation. In stark contrast, the rotation observed in the present study is induced solely by electric current without temperature nor magnetic-field gradient. It should be also emphasized that direction of the rotations detected in the earlier studies depend on the magnetic-field direction, indicating that the origin of the skyrmion lattice rotations are related to the time-reversal-variant scalar-spin chirality. On the other hand, independent rotation direction of the magnetic field direction observed in the present study indicates that the rotation is due to the time-reversal-invariant term, the dissipative tensor. Thus, we conclude that the origin of the skyrmion lattice rotation in this study is essentially different with those in the earlier studies. The averaged rotation angles for the six skyrmion reflections are plotted in Fig. 4i, j for the left-edge and right-edge parts, respectively. A counter-rotating behavior that is clearly antisymmetric around *j* = 0 is observed for the two edge positions with a rotation angle of approximately ±5 degrees for *j* = 2.7 MA m^−2^ at *T* = 28.6 K and *B*_ext_ = ±0.2 T. Hence, we conclude that the peak broadening is mainly due to a spatially inhomogeneous rotation of the skyrmion lattice, with counter-rotating behavior for the left-edge and right-edge. It should be further noted that the sum of absolute rotation angle at each edge is in semiquantitative agreement with the overall broadening observed from imaging across the whole sample (Fig. 1c), supporting our conclusion.

## Discussion

Plausibly related to the present observation is a recent magneto-optical Kerr study of the motion of single skyrmions under current flow in a CoFeB-based trilayer, where the velocity of the skyrmion near the edge of the sample is reported to be 40% less than that at the center of the sample^38^. Assuming similar slow skyrmion flow near the edges of the bulk MnSi sample, we expect that a shear flow of the skyrmion-lattice domains may appear in our sample where the velocity of the skyrmion-lattice domains is faster in the middle region only for a short time after applying electric current. After some time, the rotational torque generated by the shear flow (Fig. 4k) will be balanced by the restoring force of the skyrmion lattice, and hence the lattice may be deformed, resulting in the counter-rotating behavior observed in the present study. In other words, the skyrmion lattice flows with a shear component plausibly due to significant friction near the edges. From the rotation direction of the skyrmion lattice relative to the direction of the electric current as shown in Fig. 4c–h, we can deduce that the skyrmion motion in MnSi is along the charge current not the electron current. This is in agreement with the conclusion based on the topological Hall resistivity measurement that macroscopically the skyrmions in MnSi drift in the same direction as the electric current flows (K. Everschor, private communication^32^).

As discussed above, the slower skyrmion flow, or in other words, friction effect, near the sample edges may be the key for the observed skyrmion-lattice rotation. We speculate that such slower flow of the skyrmions is related to the enhanced pinning near the edges. It has been reported that the magnetic skyrmions are confined inside the sample by the confining potential, resulting in stronger pining effect acting on the skyrmions near the edge, as discussed in ref. ^39^. We also expect that in reality the sample would have inevitable structural/chemical disorder at the sample edge induced by the cutting and polishing procedure, which results in larger number of pinning sites near the sample edge. Thirdly, as a more physical origin of enhanced pinning, we may expect an effect of additional Dzyaloshinskii-Moriya (DM) interactions originating from the lack of inversion symmetry at surface; for Bloch-type skyrmions, such as those in MnSi, the surface DM interaction would be added on the intrinsic (bulk) DM interaction, and hence the number of skyrmions might increase near sample edges, resulting in enhanced pinning effect. At present, however, we are not sure which of these effects, if any, are deterministic for the friction effect near edges inducing skyrmion lattice rotation observed in the present study. Further study, in particular from the theoretical viewpoint, is definitely necessary to elucidate this apparently interesting issue.

Another remaining issue is why the friction effect was seen in such a large (mm sized) region away from the sample edge. In the simple particle model, where skyrmions are dense and move randomly, this edge effect works only near the edge (~1 nm). We speculated that the lattice formation is the key to propagate the friction effect in the mm sized sample. For quantitative understanding of the generation of a shear stress deeper inside the sample, however, a numerical simulation including interskyrmion interactions that takes into account friction amongst the skyrmions, as well as the robustness of their formed triangular lattice, is required.

Lastly, we comment on the effect of the thermal gradient. Despite our effort to minimize the thermal gradient, a small but finite gradient remained along the electric-current direction. This thermal gradient was pointed out as the origin of the skyrmion-lattice rotation in ref. ^31,40^ However, since the rotational motion due to the spin transfer torque under the thermal gradient parallel to the electric current should depend on the macroscopic magnetization direction, one expects that the sign of the rotation angle is switched when the external magnetic field is reversed. We clearly found that the rotation angle at each edge does not change upon inversion of the magnetic-field direction, which excludes the possibility of the remaining thermal gradient giving rise to the skyrmion-lattice rotation in the present setup.

In summary, we have used SANS to study skyrmion-lattice motion in bulk MnSi under electric current flow in a thermally homogenous condition. The azimuthal width of the skyrmion-lattice peaks shows significant broadening above a threshold current density *j*_t_ ~ 1 MA m^−2^. We show that this peak broadening originates from a spatially inhomogeneous rotation of the skyrmion lattice, that shows opposite senses of rotation at the sample edges. An intriguing memory effect was also observed for the peak broadening and the corresponding rotation of the skyrmion lattice, indicating that the driven skyrmion lattice considerably deforms due to the friction near the sample edges.

## Methods

### Single crystal growth and sample quality.

MnSi single crystal was grown by the Czochralski method. The sample quality is checked by the electric resistivity measurement. The helical ordering anomaly was detected at ~29 K. The residual resistivity ratio *R*_300K_/*R*_1.9K_ is ~53, which is the same order of magnitude as those of the samples used in the earlier works^31,32^. Details of electric resistivity measurement can be found in the Supplementary Note 1.

### Small angle neutron scattering under electric current flow.

SANS experiments were carried out at NG7 SANS instrument in National Institute of Standards and Technology and SANS-II SANS instrument in Paul Scherrer Institut. The single-crystal-MnSi samples were cut in a rectangular shape of 1.4 mm (width) × 7.5 mm (height) × 0.4 mm (thickness) for NG7. The incident neutron wavelength was *λ*_i_ = 6 Å with Δ*λ*/*λ*_i_ = 14%. Full details of our experimental setting can be found in the Supplementary Note 2. The analysis methods of the SANS data and the other SANS data are shown in the Supplementary Note 3 and 4, respectively. To further limit any temperature inhomogeneity in the measured region, and also to check the sample position dependence, only a small part of the sample was illuminated by using a very narrow beam of 2.0 mm (width) × 1.0 mm (height). An electric current up to 2 A (*j* = 3.6 MA m^−2^) was applied along the [0 0 1] direction. For such a large electric current, the sample temperature deviates from the sensor temperature slightly due to self-heating. Hence, we estimated the sample temperature using the ordering temperature of the zero-field helical phase; the maximum difference between the sensor and sample temperatures was 0.16 K at *j* = 2.7 MA m^−2^. The temperature gradient along the current-flow direction was estimated by measuring the position dependence of the ordering temperature, and was confirmed to be less than 0.035 K mm^−1^ at the sample region investigated in the present study under *j* = 2.7 MA m^−2^. The thermal gradient perpendicular to the current-flow direction was also confirmed to be approximately 0.03 K mm^−1^ under *j* = 2.7 MA m^−2^. This is at least one order of magnitude smaller than in the previous experiment^31^. In Supplementary Note 5 and 6, the detailed methods how to determine the thermal gradient inside the sample are shown. The sample mount was installed into a horizontal-field magnet with the magnetic field applied along [1 –1 0] parallel to the incident neutron beam. *B*_ext_ stands for the external magnetic field without correcting for the magnetic permeability of the MnSi sample.

### Plastic deformation of the skyrmion lattice.

From the peak width of the skyrmion reflection, the domain size is small with less than 10 skyrmion unit cells. It is expected that the skyrmion-lattice domain deforms with dislocation slipping along the domain boundary. This kind of deformation is called plastic deformation^41^. Thus, in this paper, we use the terminology of ‘plastic deformation’ to explain the observed shear distortion of the skyrmion lattice. Details of the model of the plastic deformation can be found in the Supplementary Note 7.

## Supplementary Material

Supp1

## Data Availability

The data that support the plots within this paper and other findings of this study are available from the corresponding author upon reasonable request.
