# Supplementary material for: Deformation of the moving magnetic skyrmion lattice in MnSi under electric current flow
Source: Commun Phys. Author manuscript; Available in PMC 2024 Jun 24. (PMC11194748; doi:10.1038/s42005-019-0175-z)
Supplement: Supp1 [file NIHMS1613442-supplement-Supp1.pdf]

Supplementary Note 1. MnSi single crystal

MnSi single crystal was grown by the Czochralski method. The sample quality is checked by the electric resistivity measurement shown in supplementary figure 1. A four-point probe method with electrodes of Ag-epoxy paste was used to measure the resistivity. A sample of 1.0 (width) mm $\times$ 7.4 mm (length) $\times$ 0.9 mm (thickness) was used and the electrodes distance between the voltage terminals was 2.7 mm. The helical ordering anomaly was detected at $\sim$ 29 K. The residual resistivity ratio $R_{300\mathrm{K}}/R_{1.9\mathrm{K}}$ is $\sim$ 53, which is the same order of magnitude as those of the samples used in the earlier works [1,2].

Supplementary Note 2. Details for small-angle neutron scattering experiment under electric current flow

To perform the small angle neutron scattering (SANS) experiment under the electric current flow with suppressing thermal gradient as small as experimentally achievable, we prepared two settings as shown in supplementary figure 2. In the experiment at SANS-II in Paul Scherrer Institut, we used the setting A in supplementary figure 2a. The whole sample mount was made of oxygen-free Cu, except for the neutron window where a single crystal sapphire was used. The MnSi sample was mounted on the sapphire plate using CYTOP glue. A cernox thermometer was directly glued on the surface of the sample. The sample size is 1.2 mm (width) $\times$ 8 mm (length) $\times$ 1 mm (thickness). Ag-epoxy paste and thin Cu plates were used as electrodes in this setting. The electric current and magnetic field were applied along the [0 0 1] and [1 -1 0] directions, respectively. All

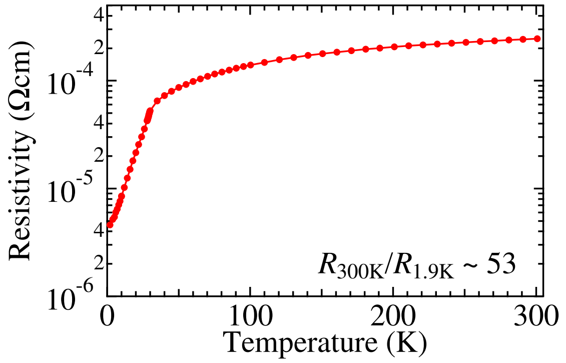

Supplementary Figure 1 | Temperature dependence of the electric resistivity of the MnSi sample.

the SANS data in the setting A were collected by integrating the intensity with rocking the sample for ± 5 degrees at 1 degree interval around the vertical axis. In the SANS-II experiment, almost the whole sample was illuminated by the incident neutron beam with the size of 1.2 mm (width) × 8 mm (height). The incident neutron wavelength was selected using the velocity selector as λ_i = 7.08 Å at SANS-II.

For the experiment at NG7 in National Institute of Standards and Technology, the setting B in supplementary figure 2b was used. The MnSi sample was cut into the rectangular shape of 1.4 mm (width) × 7.5 mm (length) × 0.4 mm (thickness) for the NG7 experiment. To reduce the contact resistance, 50 nm gold film was deposited on the electrode area of the sample surface. The thin Cu plates were indium soldered as electrodes on the Au deposited area. The total resistance including the sample and Cu current leads was less than 50 mΩ at room temperature. The sample was mounted on a sapphire plate, with a cernox thermometer glued directly to its surface using CYTOP glue. The applied electric-current and magnetic-field directions were the same as those in

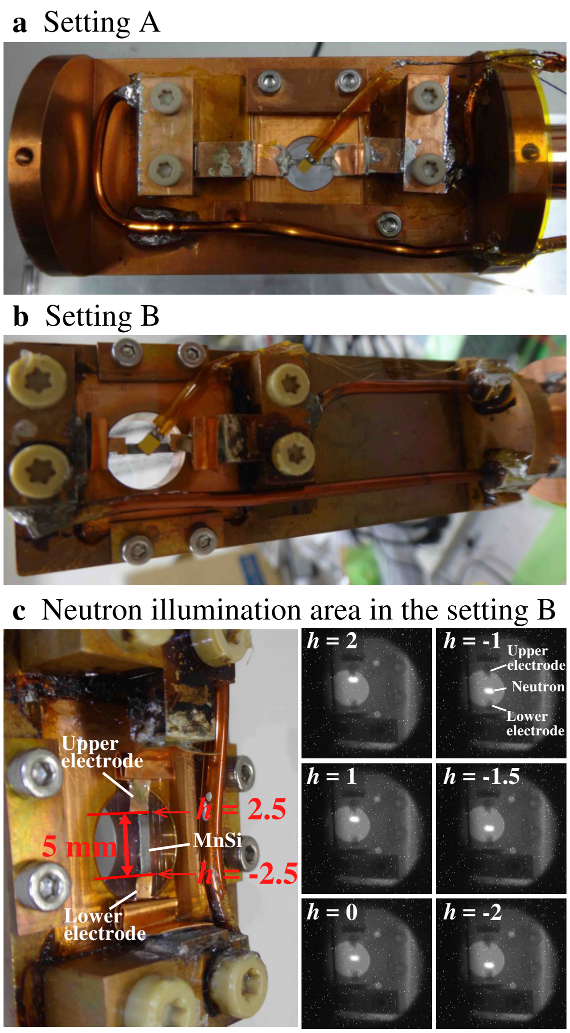

Supplementary Figure 2 | Settings of the small-angle neutron scattering experiments. **a**,**b**, Setting A (**a**) and B (**b**) were prepared for the small-angle neutron scattering experiments under the electric current flow at SANS-II in Paul Scherrer Institut and NG7 in National Institute of Standards and Technology, respectively. **c**, Height position (h) of the incident neutron beam and the corresponding neutron illumination area on the sample in the setting B. Photograph shows the position of the MnSi sample and the Cu electrodes. The center position of the sample was defined as h = 0. The edges of the upper and lower Cu electrodes are illuminated by the neutron beam at h = 2.5 and -2.5 mm, respectively. The neutron illumination area was taken by a neutron camera.

setting A. The temperature dependence data were measured after field-cooling at the target values of the magnetic field and the current density. The temperature fluctuation in the measurements was less than 0.01 K for this setting. All the SANS data shown in the setting B were collected with a fixed sample-rotation position without rocking the sample. The incident neutron wavelength was selected as λ_{i} = 6 Å for the NG7 experiment.

To correctly estimate the temperature gradient inside the MnSi sample along the electric-current-flow direction in the setting B, we measured the sample-position dependences of the ordering temperatures of the helical and skyrmion phases. The incident neutron beam was shaped to be 2.0 mm (width) $\times$ 1.0 mm (height) by an aperture. In supplementary figure 2c, the actual height position (h) of the incident neutron beam and the corresponding neutron illumination area are shown. In this setting, the center position of the sample was defined as h = 0. The cernox thermometer was glued on the sample surface near the center position of the sample. At h = -2.5 and 2.5 mm, the edges of the upper and lower electrodes are illuminated by the incident neutron beam, respectively. From the result of the sample-position dependence of the ordering temperature reported in the Supplementary Note 6, the h = -1.5 mm position was selected as the minimum-temperature-gradient position in the current study.

Supplementary Note 3. Data analysis method

To quantitatively analyze the observed peak broadening as a function of temperature and current density, we first obtained the azimuthal angle dependence of the scattering intensity by integrating

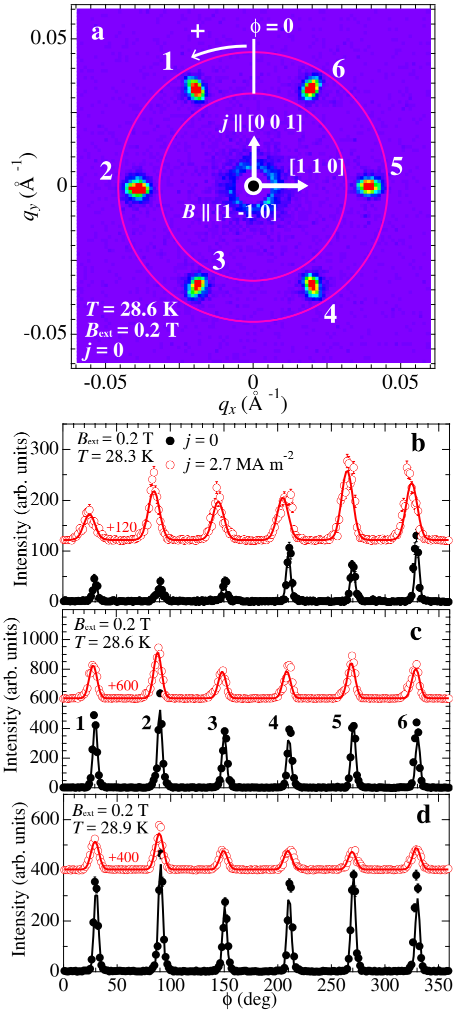

Supplementary Figure 3 | Data analysis method for the observed small-angle neutron scattering. **a**, Small-angle neutron scattering measured at T = 28.6 K and B_{ext} = 0.2 T under j = 0 in the setting B. The position indicated by the white line was defined as ϕ = 0 and the counterclockwise direction was defined as + ϕ. The numbers of 1 to 6 indicate the corresponding skyrmion reflections in **c** and supplementary figure 5. **b-d**, The azimuthal angle dependences of the intensity integrated along the radial direction between the two red circles in **a** at T = 28.3 K (**b**), at T = 28.6 K (**c**), and at T = 28.9 K (**d**), respectively. Black filled and red open circles stand for the data at j = 0 and 2.7 MA m^{-2}, respectively. The solid lines show the fitting results using six Gaussian functions.

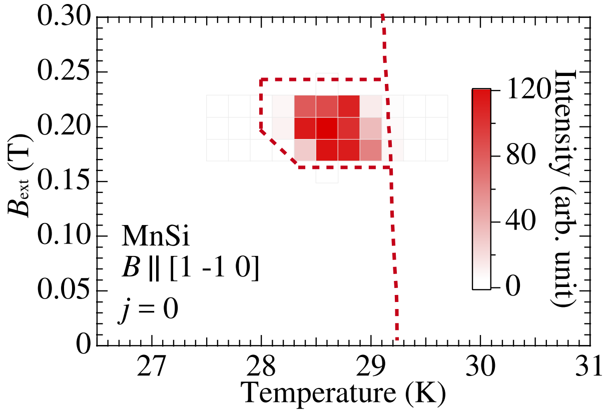

Supplementary Figure 4 | Skyrmion-phase diagram at B || [1 -1 0] obtained from the total scattered SANS intensity of the skyrmion reflections in the MnSi sample. Dotted line is guide to eye for the phase boundary.

along the radial direction between the two red circles shown in supplementary figure 3a. Typical examples of such azimuthal angle dependence are shown in supplementary figure 3b-d. Hereafter, we use 1σ standard deviation as error bar. The data in supplementary figure 3b-d were measured at T = 28.3, 28.6, and 28.9 K, respectively, at B_{ext} = 0.2 T. B_{ext} stands for the external magnetic field without the correction of the magnetic permeability of the MnSi sample. Black filled and red open circles respectively stand for the applied electric current density j = 0 and 2.7 MA m^{-2}. Here, the position indicated by the white line in supplementary figure 3a was defined as ϕ = 0. The counterclockwise direction was defined as + ϕ. The azimuthal angle dependence was further fitted to six equally distributed Gaussians and background intensity, from which the full width at half maximum (FWHM) of the peak was obtained. The black and red solid lines in supplementary figure 3b-d show the fitted curves. The total scattered SANS intensity was estimated by trapezoidal integration after subtracting the background intensity. Supplementary figure 4 shows the skyrmion-

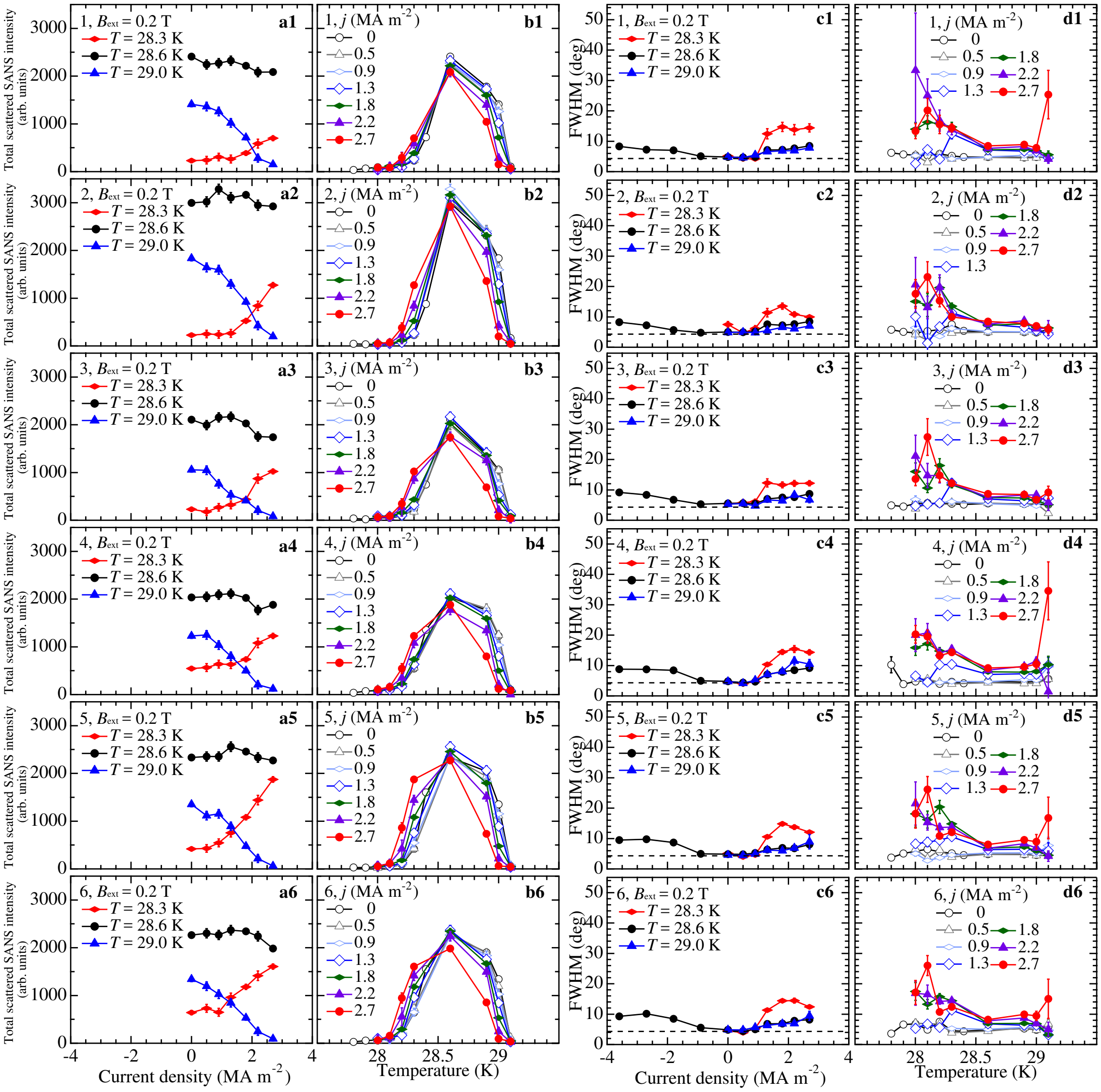

Supplementary Figure 5 | Total scattered small-angle neutron scattering intensity and FWHM obtained by the Gaussian fitting for each skyrmion reflection. **a-d**, Current-density (**a**,**c**) and temperature (**b**,**d**) dependences of the total scattered SANS intensity and peak width for each six skyrmion reflection individually indicated by the numbers of 1 to 6. The numbers indicate the corresponding skyrmion reflections in supplementary figure 3. The total scattered SANS intensity is obtained by trapezoid integration for each skyrmion reflection. The peak width is estimated by fitting six Gaussian functions with six FWHM parameters.

phase diagram at $B \parallel$ [1 -1 0] obtained by the summation of the intensities of the six skyrmion reflections. The skyrmion phase was observed in a temperature range of approximately 28 K $<$ T $<$ 29.2 K at $B_{\rm ext}$ = 0.2 T and in a magnetic-field range of 0.18 T $<$ $B_{\rm ext}$ $<$ 0.22 T at T = 28.6 K. In supplementary figure 5, the total scattered SANS intensity and FWHM obtained by the Gaussian fitting for each skyrmion reflection are shown. The numbers 1 to 6 in the figure indicate the corresponding peak positions in the SANS pattern and azimuthal angle dependence shown in supplementary figure 3a,c. The temperature used in the figures is the sensor temperature, and is not the true temperature estimated from the ordering temperature of the helical phase (explained in supplementary note 5). The current-density and temperature dependence of the intensity and peak width for each peak individually is identical with those in Fig. 3 of the main text. Hence in the main text, to improve the precision of the intensity and peak width values, we used the summation of the total scattered SANS intensity and the fitting result using single FWHM parameter. In Fig. 3b of the main text, the peak width at 28.3 K reaches to up 15° at $j \sim$ 2.0 MA m^{-2}, and decreases at higher current density (measured up to j = 2.7 MA m^{-2}). In contrast, the peak width obtained at 28.6 K saturates to $\sim$ 9° at j = 2.7 MA m^{-2}, which is smaller than those at 28.3 K. The broadening effect was also observed using the thicker sample with the large illumination area using the SANS-II instrument. The open circles in Fig. 3b of the main text stand for the current dependence of the peak width similarly estimated from the thicker sample result. Although the initial ($j = 0$) width of the peak is different, possibly due to the larger temperature inhomogeneity originating from the large illumination region, the threshold current density and the broadening behavior above $j_{\rm t}$ are consistent with the thinner sample. This clearly indicates the reproducibility of the current-

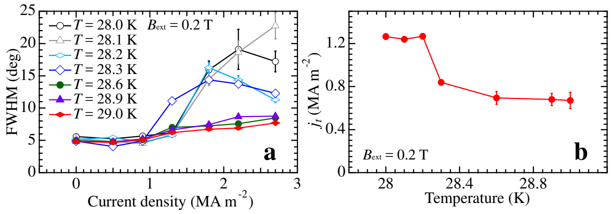

Supplementary Figure 6 | Temperature dependence of the threshold current density. **a**, Current-density dependence of the peak width of the skyrmion reflection for each temperature measured at B_{ext} = 0.2 T. **b**, The temperature dependence of the threshold current density j_{t} necessary for the motion of the skyrmion lattice. To obtain j_{t}, the current-density dependence of the peak width in **a** was fitted by the following function, $\sigma = \sigma_0 + b \times (j - j_{\mathrm{t}})$. Here, σ and j stand for FWHM and current density, respectively. σ_0, b, and j_{t} are respectively background, constant value, and threshold current density.

broadening effect, irrespective of sample size and related small temperature inhomogeneity.

Supplementary figure 6b shows the temperature dependence of the threshold current density obtained by fitting the current-density dependence in supplementary figure 6a to a combination of a constant background and a linearly increasing function. The threshold current density increases with decreasing temperature, in particular below 28.3 K. One can find that the temperature dependence of the threshold current density from the SANS data is different from that obtained by the topological Hall resistivity [2]. The reason for this difference remains unclear.

Supplementary Note 4. Current-density dependence of Q position of the skyrmion reflection

To investigate the variation of the spacing of the skyrmion lattice with temperature and current density, the reciprocal lattice position Q and peak width along $Q\hat{e}_Q$ direction of the skyrmion reflection were estimated. At first, we obtained the Q dependence of the intensity integrated along the azimuthal angle direction in the yellow colored area in supplementary figure 7a. In supplementary figure 7b, the example of the summation of the intensity of the six skyrmion reflections is shown. The total scattered SANS intensity was fitted by single Gaussian function, from which Q position and peak width along the Q were obtained. The fitting result is shown by the solid red line. In supplementary figure 7c,d, the obtained temperature dependences of the Q position and peak width along the Q direction for each current density are shown, respectively. The peak width is almost temperature and current density independent, and its value is $\sim 0.5 \times 10^{-2}$ Å^{-1} with slight increases above $T \sim 29$ K. This should originate from the decrease of the correlation length of the skyrmion lattice near the transition temperature. The temperature dependence of the Q position for each current density is negligible within the accuracy of the experiment. The Q position of the skyrmion reflection is approximately 3.9×10^{-2} Å^{-1}, from which the spacing of the skyrmion lattice is estimated as ~ 160 Å.

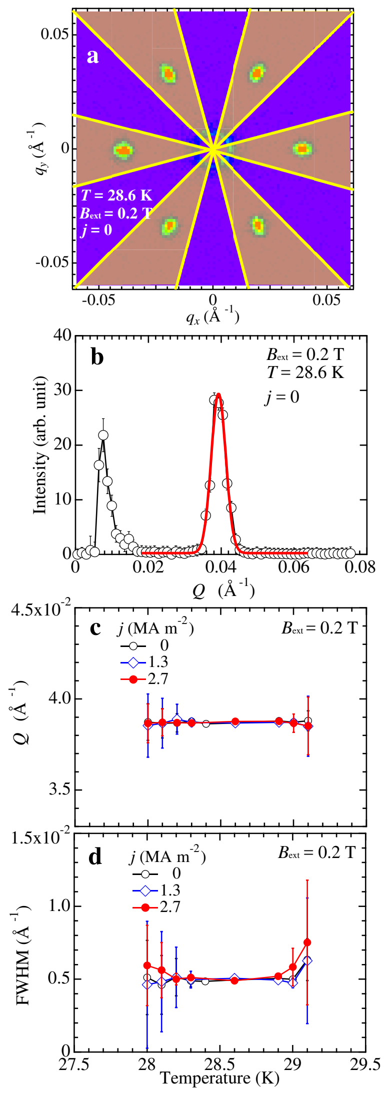

Supplementary Figure 7 | Variation of the spacing of the skyrmion lattice with temperature and current density. **a**, Example of the analysis of small-angle neutron scattering data to obtain Q dependence of the intensity integrated along the azimuthal angel direction in the yellow colored area. **b**, Q dependence of the summation of the intensities of the six skyrmion reflections. Fitting with single Gaussian function, the peak position and width were obtained. Red line shows the fitting result. **c**,**d**, The temperature dependences of the peak position (**c**) and peak width (**d**) of the skyrmion reflection for each current density measured at B_{ext} = 0.2 T.

Supplementary Note 5. SANS data in the helical phase under electric current flow and temperature-reading correction using the helical-phase-ordering temperature

Supplementary figure 8 shows the current-density dependent SANS data in the helical phase measured in the setting B. Examples of raw SANS patterns at T = 28.6 K and B_{ext} = 0 under j = 0 and 2.7 MA m^{-2} are respectively shown in supplementary figure 8a,b. By using the analysis procedure described in supplementary note 3, the temperature dependence of the total scattered SANS intensity for each current density was obtained. To determine the ordering temperature of the helical phase, the temperature dependence of the intensity was fitted to the following equation to determine the critical temperature,

$$I = a \times [(T_{\mathrm{c}} - T)/T_{\mathrm{c}}]^{\beta}. \tag{S1}$$

I and T stand for intensity and temperature, respectively. a, T_{c}, and β are constant value, ordering temperature, and critical exponent. The fitting result is shown in supplementary figure 8c-i, from which the ordering temperature T_{c} for each current density was estimated. In supplementary figure 8j, the current-density dependence of the ordering temperature of the helical phase is summarized.

The ordering temperature of the helical phase read by the thermometer monotonically decreases with increasing the current density. Here, we assume that the ordering temperature of the helical phase is insensitive to the applied electric current flow at least near the current density $\sim$ 1 MA m^{-2}. This is because the threshold current density necessary for the domain motion of the helical ordering would be above several GA m^{-2}, which is much higher than the current density

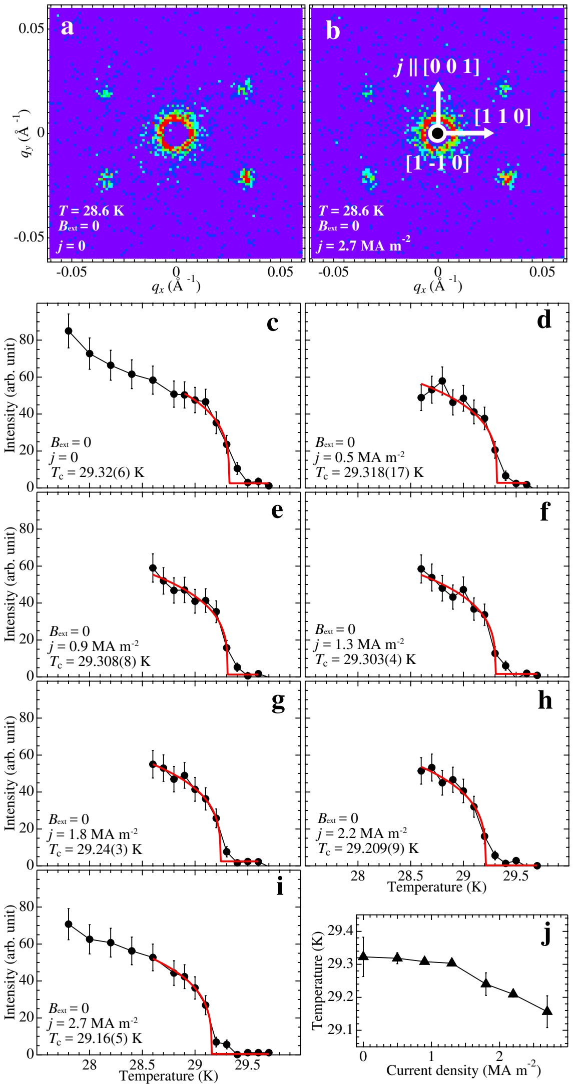

Supplementary Figure 8 | Current-density dependent small-angle neutron scattering data to determine the ordering temperature of the helical phase. **a**,**b**, Small-angle neutron scattering data in the helical phase at T = 28.6 K and B_{ext} = 0 under j= 0 (**a**) and 2.7 MA m^{-2} (**b**) measured in the setting B. **c**-**i**, Temperature dependence of the total scattered SANS intensity of the helical reflection at j = 0 (**c**), 0.5 (**d**), 0.9 (**e**), 1.3 (**f**), 1.8 (**g**), 2.2 (**h**), and 2.7 MA m^{-2} (**i**), respectively. Red lines show the fitting results using equation (S1). **j**, Current-density dependence of the ordering temperature of the helical phase. Here, we use the fitting error as error bar.

in the present experimental setting. This allows us to convert the sensor temperature to the true sample temperature under electric current using the ordering temperature of the helical phase. The maximum temperature difference between the sample and sensor temperatures was 0.16 K at j = 2.7 MA m^{-2} in the setting B. The temperatures in Fig. 3d-f of the main text are the ones estimated as described above. In the setting A, the maximum temperature difference between the sample and sensor temperatures was approximately 0.9 K at j = 1.67 MA m^{-2}. In the data of the thicker sample in Fig. 3b of main text, the true sample temperature estimated here is used.

We also investigated the current-density dependence of the peak width along the azimuthal direction of the helical reflection. For this measurement, the setting A was used. In supplementary figure 9a,b, the four magnetic reflections along [1 1 1] direction measured at T = 28.6 K, $B_{\rm ext}$ = 0 and j = 0, and at T = 27.7 K, $B_{\rm ext}$ = 0 and j = 1.67 MA m^{-2} are shown, respectively. Here, the sensor temperature is used. In these figures, the background intensity was subtracted. By using the analysis procedure written in supplementary note 3, the azimuthal angle dependence of the total scattered SANS intensity was obtained as shown in supplementary figure 9c,d. By the four-Gaussian fitting, the current-density dependence of the total scattered SANS intensity and peak width along the azimuthal angle of the helical reflection was obtained as shown in supplementary figure 9e,f. The peak width is less current-density dependent, which is in contrast to that of the skyrmion reflection. From this result, we conclude that the helical magnetic structure, as well as the underlying crystal lattice, is less sensitive to the electric current. Hence, crystal-lattice deformation is not the main origin of the significant broadening of the skyrmion reflection above $j_{\rm t}$.

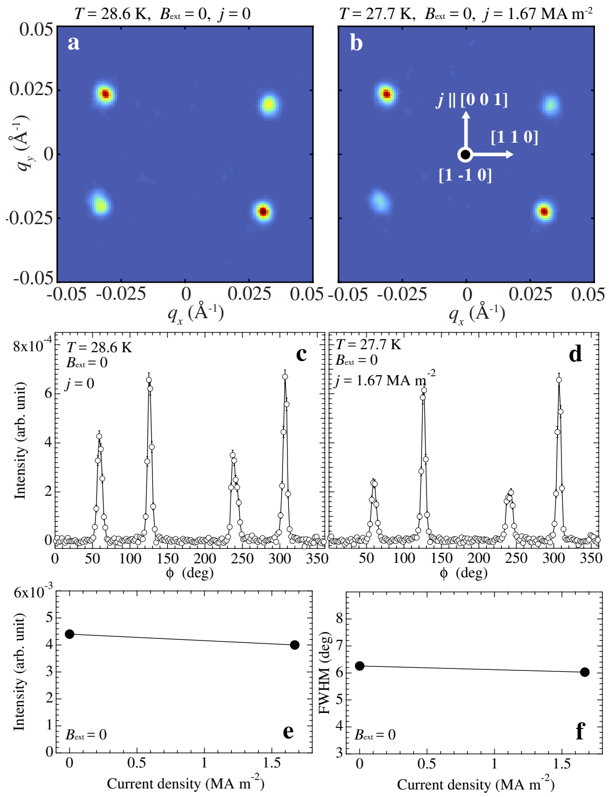

Supplementary Figure 9 | Current-density dependence of the peak width along the azimuthal direction of the helical reflection. **a**,**b**, Small-angle neutron scattering data in the helical phases ($B_{\rm ext}$ = 0) at T = 28.6 K under j = 0 (**a**), and at T = 27.7 K under j = 1.67 MA m^{-2} (**b**) measured in the setting A. Here, the sensor temperature is used. **c**,**d**, The azimuthal angle dependence of the total scattered SANS intensity at j = 0 (**c**) and 1.67 MA m^{-2} (**d**). **e**,**f**, Current-density dependence of the total scattered SANS intensity (**e**) and peak width along the azimuthal angle direction (**f**) of the helical reflection.

Supplementary Note 6. Temperature gradient measurement inside the sample

To determine the temperature gradient inside the MnSi sample along the current-flow direction (height-position direction of the sample), we measured the height-position (h) dependence of the ordering temperature in detail. Here, the temperature read by the thermometer was used. We used the ordering temperature of the skyrmion phase instead of the helical phase, because the skyrmion reflections were measured with long-time exposure and its total scattered SANS intensity was larger than that of the helical reflection. However, to use the ordering temperature of the skyrmion phase, we need to confirm that the transition temperature of the skyrmion phase also does not depend on the electric current. The temperature dependence of the intensity of the skyrmion reflection was fitted by using the equation (S1) as shown in supplementary figure 10a-g, and the ordering temperature of the skyrmion phase read by the thermometer was estimated. Red curves show the fitting results. In supplementary figure 10h, the current-density dependences of the ordering temperatures of the helical and skyrmion phases read by the thermometer are summarized. Here, the temperature differences of the ordering temperatures individually obtained by subtracting the T_{c} values at j = 0 for the skyrmion (black circle) and helical (red triangle) phases are shown, respectively. The temperature difference of the skyrmion phase also monotonically decreases with increasing the current density, consistent with the helical phase in the accuracy of the experimental error bar. Thus, we conclude that the change of the transition temperature of the skyrmion phase by applying the electric current is negligible, and the observed decrease of the ordering temperature is also due to the temperature difference between the sample and sensor temperatures.

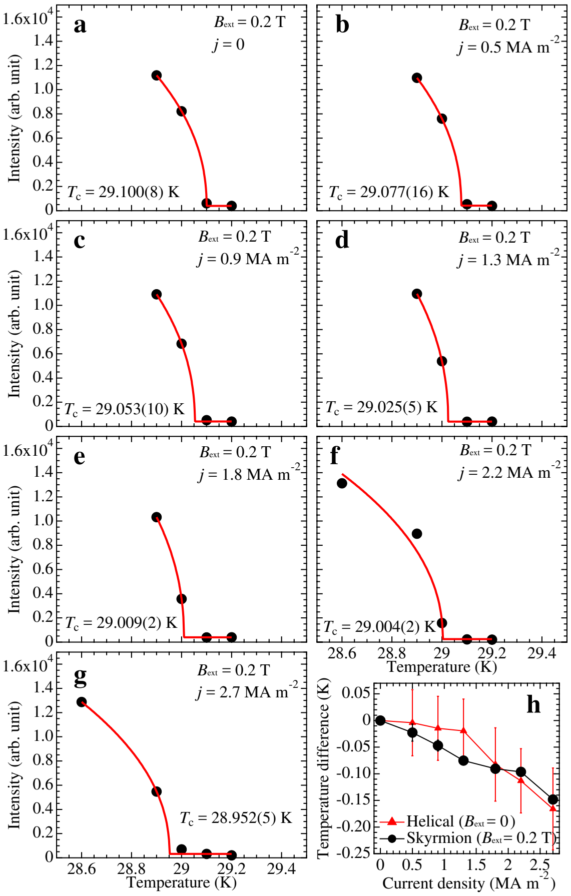

Supplementary Figure 10 | Current-density dependent small-angle neutron scattering data to determine the ordering temperature of the skyrmion phase. **a-g**, Temperature dependence of the total scattered SANS intensity of the skyrmion reflection at j = 0 (**a**), 0.5 (**b**), 0.9 (**c**), 1.3 (**d**), 1.8 (**e**), 2.2 (**f**), and 2.7 MA m^{-2} (**g**), respectively. Red lines show the fitting results using the equation (S1). **h**, Current-density dependences of the temperature difference of the ordering temperatures individually obtained by subtracting the T_c value at j = 0 for the skyrmion (black circle) and helical (red triangle) phases. Here, we use the fitting error as error bar.

Supplementary figure 11a-j show the temperature dependence of the total scattered SANS intensity of the skyrmion reflections at $B_{\rm ext}$ = 0.2 T under j = 0 and 2.7 MA m^{-2} for each h position of the incident neutron beam. Here, again, the temperature read by the thermometer was used. In supplementary figure 2c, the corresponding neutron illumination area is shown. The ordering temperature read by the thermometer was obtained by fitting the data to the equation (S1). In supplementary figure 11k,l, the height-position dependences of the temperature differences of the ordering temperatures read by the thermometer individually obtained by subtracting the $T_{\rm c}$ values at h = -2 mm under j = 0 (blue triangle) and 2.7 MA m^{-2} (red circle) and its schematic illustration are summarized, respectively. At j = 0, the temperature difference was nearly equal 0 up to h = 0. The maximum temperature difference is $\sim$ 0.09 K observed at h = 2 mm. This result indicates that the temperature gradient in the sample from h = -2 to 0 is approximately 0 at j = 0. In contrast, at j = 2.7 MA m^{-2}, the maximum temperature difference becomes twice $\sim$ 0.18 K at h = 2 mm, while the temperature difference at h = -1 mm is still negligible in the accuracy of the experiment. To estimate the upper limit of the temperature gradient in the sample from h = -2 to -1 mm at j = 2.7 MA m^{-2}, we interpolated the temperature difference between h = 0 and h = -2 mm, estimated that the temperature is less than $\sim$ 0.035 K mm^{-1} at j = 2.7 MA m^{-2}. This temperature gradient value is much smaller than $\sim$ 0.2 K mm^{-1} at $j \sim$ 2 MA m^{-2} of the earlier work [1]. From this result, we selected the h = -1.5 mm as the minimum-temperature-gradient position in the present study.

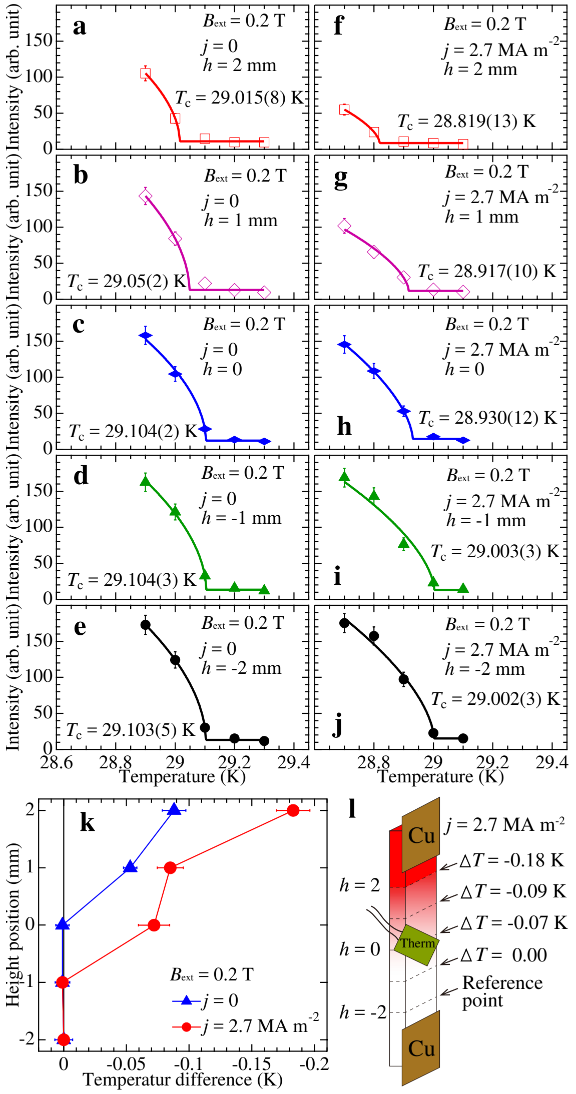

Supplementary Figure 11 | Temperature gradient inside the MnSi sample along the current-flow direction. **a-j**, Temperature dependence of the total scattered SANS intensity of the skyrmion reflection at B_{ext} = 0.2 T for each height (h) position under j = 0 and 2.7 MA m^{-2}. The ordering temperature was obtained by fitting to the equation (S1). The solid lines show the fitting results. **k**, The height-position dependence of the temperature difference of the ordering temperature individually obtained by subtracting the T_c value at h = -2 mm under j = 0 (blue triangle) and 2.7 MA m^{-2} (red circle). **l**, Schematic view of the temperature gradient inside the sample at j = 2.7 MA m^{-2}.

Supplementary Note 7. Model of the plastic deformation of the skyrmion lattice with shear flow

A naive model to explain the plastic deformation of the skyrmion lattice with the shear flow is shown in supplementary figure 12. We speculate the existence of the unrotated domain of the skyrmion lattice between the counterclockwise and clockwise rotated domains at the high electric current density ($j \geq j_t$). At the domain boundary, we also speculate the existence of the dislocations with consisting of the five-coordinated skyrmion connected to a seven-coordinated skyrmion. With these dislocations slipping along the domain boundary, the skyrmion lattice domains deform. To clarify whether this model is correct, we need to measure the sample position dependence of the rotation angle of the skyrmion lattice perpendicular to the electric-current-flow direction in near future.

Supplementary References

1. F. Jonietz, S. Mühlbauer, C. Pfleiderer, A. Neubauer, W. Münzer, A. Bauer, T. Adams, R. Georgii, P. Böni, R. A. Duine, K. Everschor, M. Garst, and A. Rosch, Science **330**, 1648 (2010).

2. T. Schulz, R. Ritz, A. Bauer, M. Halder, M. Wagner, C. Franz, C. Pfleiderer, K. Everschor, M. Garst, and A. Rosch, Nat. Phys. **8**, 301 (2012).

3. M. Janoschek, F. Jonietz, P. Link, C. Pfleiderer, P. Böni, J. Phys: Conference Series **200**, 032026 (2010).

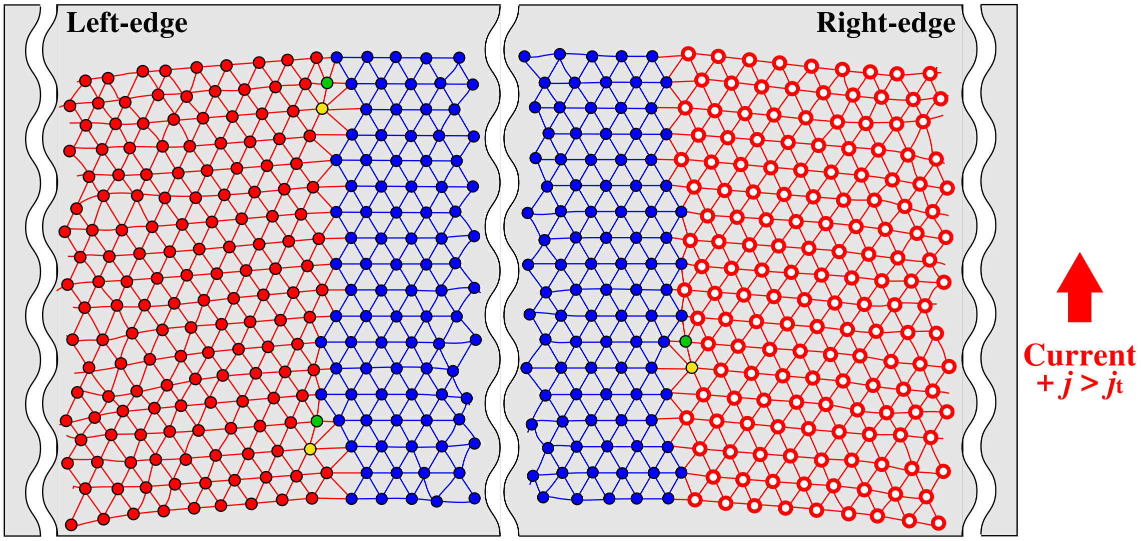

Supplementary Figure 12 | Model of the skyrmion-lattice deformation at high electric current density ($j \geq j_t$). The red filled/opened circles stand for the counterclockwise-/clockwise-rotated-skyrmion lattice, respectively. The blue filled circle is the unrotated skyrmion lattice. The dislocations with consisting of five-coordinated skyrmion connected to a seven-coordinated skyrmion are indicated by the filled green and yellow circles, respectively. To explain the short ranged correlation of the skyrmion lattice (less than 10 skyrmion unit cells) measured by small angle neutron scattering (this study) and the cold neutron diffraction experiments[3], the distortion of the skyrmion lattice are intentionally drawn.
